# Green-Synthesized MgO Nanoparticles: Structural Insights and Antimicrobial Applications

**DOI:** 10.3390/ijms26189021

**Published:** 2025-09-16

**Authors:** Denisa-Maria Radulescu, Ionela Andreea Neacsu, Bodgan Stefan Vasile, Vasile-Adrian Surdu, Ovidiu-Cristian Oprea, Roxana-Doina Trusca, Cristina Chircov, Roxana Cristina Popescu, Cornelia-Ioana Ilie, Lia-Mara Ditu, Veronica Drumea, Ecaterina Andronescu

**Affiliations:** 1 Department of Science and Engineering of Oxide Materials and Nanomaterials, Faculty of Chemical Engineering and Biotechnologies, National University of Science and Technology POLITEHNICA Bucharest, 011061 Bucharest, Romania; 2 National Research Center for Micro and Nanomaterials, Faculty of Chemical Engineering and Biotechnologies, National University of Science and Technology POLITEHNICA Bucharest, 060042 Bucharest, Romania; 3 Romanian Academy of Scientists, 050045 Bucharest, Romania; 4 Research Center for Advanced Materials, Products and Processes, National University of Science and Technology POLITEHNICA Bucharest, 060042 Bucharest, Romania; 5 Department of Materials Science, Faculty of Materials Science and Engineering, Transilvania University of Brasov, 29 Eroilor Blvd., 500036 Brasov, Romania; 6 Department of Bioengineering and Biotechnology, Faculty of Medical Engineering, National University of Science and Technology POLITEHNICA Bucharest, Polizu 1-7, 011061 Bucharest, Romania; 7 National Institute for R&D in Physics and Nuclear Engineering Horia Hulubei, Department of Life and Environmental Physics, Reactorului 30, 077125 Magurele, Romania; 8 Department of Botany and Microbiology, Faculty of Biology, University of Bucharest, 060101 Bucharest, Romania; 9 S.C. Biotehnos S.A., Gorunului 3-5, 075100 Otopeni, Romania

**Keywords:** green synthesis, magnesium oxide nanoparticles, orange peel extract, phytochemical synergy, biocompatibility, antimicrobial activity, antibiofilm

## Abstract

Magnesium oxide nanoparticles, or MgO NPs, have garnered a lot of attention because of their exceptional stability, biocompatibility, and antibacterial properties. However, many of the green production methods used today have limited mechanistic knowledge and low reproducibility. In order to get over these challenges, we created a standardized and environmentally friendly process for producing MgO NPs using orange peel extract, a naturally occurring biowaste source rich in phytochemicals that acts as a stabilizing and reducing agent. Active precursor alteration during synthesis was clearly shown by X-ray diffraction (XRD) and thermal analysis (TGA-FTIR), while imaging techniques showed extremely crystalline cubic-phase MgO nanoparticles that were about 9 nm in size. The NPs displayed an irregular shape between 10 and 40 nm and a positive surface charge of +11.74 mV. Terpenoids, polymethoxyflavones, fatty acids, and sugars all work in collaboration with direct nucleation, regulate particle growth, and stabilize the nanoparticles, according to GC-MS analysis. The MgO NPs showed remarkable cytocompatibility in biology, preserving >80% viability in fibroblast and osteoblast cell lines while causing distinct metabolic regulation in osteoblasts without changing the shape of the cells. Consistent moderate activity against a variety of pathogens was confirmed by antimicrobial and antibiofilm assays, with special effectiveness against *Gram-positive bacteria* and *Pseudomonas aeruginosa* biofilms. This study shows that these MgO NPs have good biocompatibility and antimicrobial qualities, indicating the need for more research for possible biomedical applications. It also clarifies the molecular role of phytochemicals in nanoparticle formation and provides a repeatable green synthesis pathway.

## 1. Introduction

Nanomaterials are now considered pioneering agents in the fields of electronics, energy storage, environmental remediation, and biomedical engineering due to the quick development of nanotechnology [1]. However, because of their reliance on hazardous chemicals, high energy requirements, and unsustainable practices, current synthesis methods for inorganic metal oxides present serious environmental and safety concerns [2]. Conventional chemical methods usually involve complicated procedures requiring high temperatures, advanced equipment, and vacuum conditions, as well as reducing agents, organic solvents, metal salts, and capping agents [3]. Similarly, physical synthesis techniques require sophisticated machinery with considerable energy consumption, mechanical crushing, and harsh environments.

Through the use of harmful substances and heavy metals that linger in environmental systems, these traditional methods have increased biological and environmental concerns, affecting both ecological integrity and human health. Thus, scientists have concentrated on creating environmentally friendly substitutes for the production of nanoparticles. Green nanotechnology overcomes these constraints by using ecologically friendly techniques that reduce damage to ecosystems and human health [4]. Utilizing biological systems and natural resources, environmentally friendly approaches produce safer nanomaterials while lowering the dangers associated with conventional methods [5]. Green synthesis or biosynthesis eliminates harmful chemicals and requires few reaction conditions, such as high temperature, high pressure, or excessive energy [6]. The objective of this strategy is to lessen waste production and promote sustainable advancement in nanotechnology. In order to make the process more convenient, cost-effective, and ecologically friendly [7], this technology uses non-toxic reagents from plant substrates (leaves, stems, fruits, flowers, roots, bark), microorganisms (bacteria, fungi, algae), and biomolecules (DNA, proteins, enzymes, vitamins) [8,9,10].

Due to their distinct physicochemical characteristics and biocompatibility, MgO NPs have attracted remarkable interest in biomedical engineering among the nanomaterials being studied. Metal oxides have a variety of functions, variable band gaps, and high surface-to-volume ratios [1]. In particular, MgO nanoparticles have a large surface area, unique crystal structures, improved ionic character, and oxygen vacancies, which allow for easy interaction with biological systems [11].

Magnesium’s biological relevance makes MgO nanoparticles more appealing for applications in biomedical applications. In this direction, it is well known that magnesium, the second most prevalent intracellular cation, is an essential cofactor in more than 300 enzymatic processes, such as protein synthesis and energy metabolism. There is equal distribution between soft tissues and bones, according to scientific research [12]. MgO can balance degradation, control pH, and improve biomaterial compatibility because of its biocompatibility [13].

Due to their chemical, biological, and physical characteristics, such as their antibacterial activity, non-toxicity, thermal stability, low dielectric constant, low refractive index, and exceptional luminescence, MgO NPs exhibit enhanced versatility [14,15]. From refractory additives to drug transporters, antimicrobial agents, and hyperthermia-based cancer therapy vehicles, MgO NPs allow for a wide range of uses. In biomedicine, they exhibit antioxidants and antidiabetic properties and are used in drug delivery, tissue engineering, bone regeneration, stomach relief, and bioimaging [11,16,17,18]. The growth of nanowires, nanoflakes, nanorods, nanodisks, nanoflowers, and nanosheets is all made possible by the structural adaptability of MgO NPs [14,19,20].

In MgO synthesis, achieving high yield, homogeneity, repeatability, controlled aggregation, and desired shape is still difficult and demands environmentally friendly methods [21,22]. Although there are hydrothermal, sol-gel, co-precipitation, and chemical gas phase techniques, they use hazardous chemicals and strong acids or bases, which can harm the environment [23]. Phytochemicals found in plant extracts break down metal ions to create nanoparticles. Metal oxide reduction is aided by functional amino groups, and metal ions are further reduced by ambient oxygen or degrading phytochemicals. Particle agglomeration is then controlled by electrostatic attraction, which also stabilizes phytochemicals and mediates oxide formation [24,25].

The abundance of macronutrients (sugars, potassium, dietary fiber, vitamins) and metabolites (flavonoids, coumarins, alkaloids, phenolic acids, limonoids, and carotenoids) in orange waste makes it particularly promising [26]. Benefits of using orange peel extract for biogenic MgO production include its economic viability, biocompatibility, and convenience in green chemistry frameworks. Agricultural waste can be converted into useful resources by using orange peel waste as a bio-reducing agent, which addresses both economic efficiency and environmental sustainability. In order to achieve controlled particle size and morphology, naturally occurring bioactive chemicals perform the dual roles of stabilizing and reducing agents during nanoparticle production [27]. By offering natural buffering that controls pH and ionic strength, the extract promotes regulated precipitation, resulting in uniform size distribution and improved surface qualities [28].

By using the intricate phytochemical diversity of orange peel extract for precisely controlled MgO nanoparticle formation, this work presents a new paradigm in green nanotechnology. Beyond conventional single-agent green synthesis techniques, the novel method coordinates the actions of terpenoids, polymethoxyflavones, fatty acids, and derivatized sugars to produce hitherto unheard-of structural homogeneity and functional performance. This work provides the first thorough understanding of multi-pathway nanoparticle production in plant-mediated synthesis and indicates active precursor transformation through a thorough mechanistic explanation. The resultant MgO NPs have remarkable dual functionality, addressing important shortcomings in existing green synthesis techniques by exhibiting targeted antimicrobial efficacy against *Gram-positive* pathogens and biofilm matrices while maintaining superior biocompatibility (>80% cell viability).

Considering the growing use of MgO NPs in biomedicine and the environmental benefits of green synthesis, it is imperative to create new sustainable methods. The primary objective of this research is to create a standardized, repeatable, and environmentally friendly synthesis protocol for MgO NPs using orange peel extract as a multipurpose reducing, chelating, and stabilizing agent. It also aims to thoroughly assess the physicochemical characteristics and biological performance of these particles for tissue engineering and antimicrobial applications. By examining green synthesis with citron peel extract and contrasting it with its conventional counterparts, this study fills a knowledge gap. This study promotes ecologically friendly production techniques for biomedical applications while advancing sustainable nanotechnology and offering insight into the connection between synthesis processes and nanoparticle characteristics.

## 2. Results

### 2.1. Thermogravimetric Analysis Results

**Figure 1:**
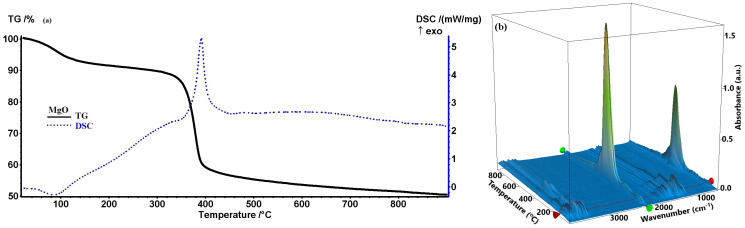
Thermogravimetric analysis of MgO (**a**); FTIR 3D diagram of the evolved gases (**b**).

As shown in Figure 1, the MgO precursor powder sample exhibits a mass loss of 8.49% up to 200 °C, accompanied by an endothermic effect with a minimum at 86.3 °C, corresponding to the loss of surface-adsorbed water molecules. Partial dehydration of Mg(OH)_2_ should be excluded at this temperature, as the endothermic transformation to MgO occurs around 330 °C [29]. For this sample, the mass loss up to 200 °C is larger than for the other samples, and the FTIR 3D diagram also indicates elimination of important CO_2_ quantities. This might originate from physically adsorbed CO_2,_ but also from the decomposition of some phytocompounds with carboxylic moieties. After 200 °C, a gradual mass loss occurs, with a slightly exothermic effect attributed to the partial oxidation of organic compounds capping the NPs, as the FTIR of the evolved gases indicates the presence of water and carbon dioxide only. After 350 °C, the sample rapidly loses mass, 35.23% up to 460 °C, with the associated effect being strongly exothermic and sharp, with a maximum at 391.6 °C. This effect indicates a burning of the organic part, which generates the high amount of carbon dioxide as indicated in the FTIR 3D diagram. Surprisingly, no carbon monoxide traces can be found in the evolved gases of this sample. The absence of detectable nitrate decomposition products (e.g., NO_2_ or NO) and the increased residual mass strongly suggest that the metal nitrates had already undergone reaction with the organic compounds in the orange extract before calcination.

After 460 °C, the sample continues to slowly lose mass, around 5.85% up to 900 °C, with the corresponding effect on the DSC curve being broad and slightly exothermic, with a maximum at 607.6 °C associated with the burning of the carbon residue in the sample. The white residual mass represents 50.43% at 900 °C. As in previous samples, the residual mass is much larger than the values obtained from Mg(NO_3_)_2_·6H_2_O (15.60%) or Mg(NO_3_)_2_ (26.97%) [30,31], indicating the successful reaction of the orange peel extract with the magnesium nitrate.

### 2.2. X-Ray Diffraction (XRD) Analysis Results

**Figure 2:**
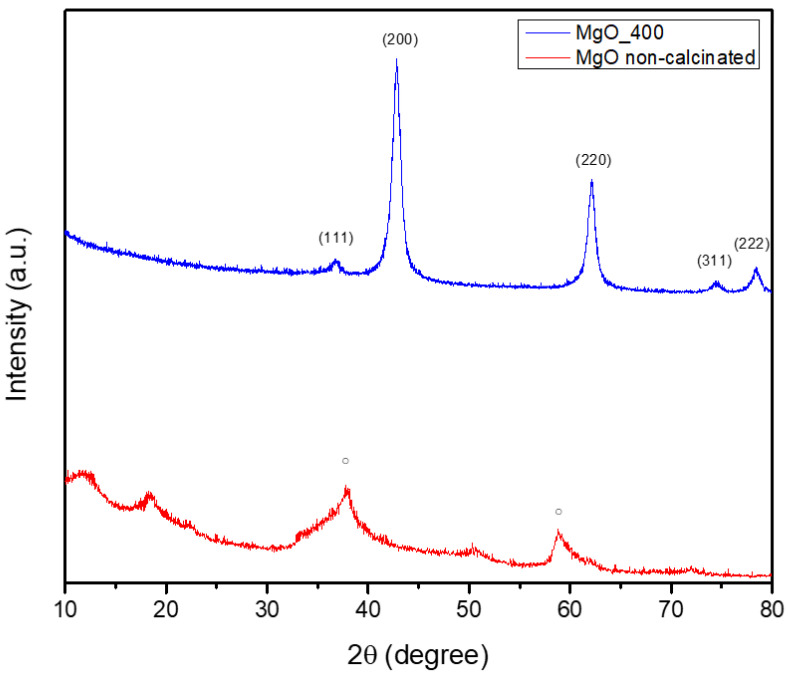
X-ray diffractogram performed on a sample of green-synthesized MgO NPs: red color—untreated MgO powder where ○—Mg(OH)_2_; and blue color—calcinated MgO at 400 °C.

Figure 2 shows the results of an XRD study that was used to determine the green-synthesized powder’s crystalline phase composition.

Figure 2 showcases the non-calcinated Mg(OH)_2_ as a single crystalline structure, with a hexagonal symmetry, as indicated by the database file number 96-210-1439. The diffraction pattern revealed notable peaks at approximately 2θ = 18°, 32°, 38°, 50°, and 58°, which correspond to Miller indices (001), (100), (011), (012), and (110), respectively. By heat-treating the MgO powder at 400 °C, a phase transformation occurred, resulting in the formation of MgO NPs. These NPs, matching the PDF4+ 04-016-6860 file, adopted a cubic symmetry. The diffractogram displayed prominent peaks at 2θ = 42°, 62°, 74°, and 78°, which were attributed to Miller indices (200), (220), (311), and (222), respectively. Additionally, the analysis revealed that the MgO NPs had an average crystallite size of 9.08 nm. Hence, it could be summarized that the calcination process consistently facilitates the decomposition of hydroxide and nitrate groups, ultimately producing oxide NPs. This transformation requires the elimination of volatile constituents (such as water and NOx) and the subsequent rearrangement of the remaining atoms into a stable oxide configuration. The formation of the oxide phase can induce changes in crystalline symmetry, as evidenced by the transitions observed in MgO (from hexagonal to cubic). Understanding these phenomena is essential for customizing nanoparticle properties to fit specific applications, given that both crystalline structure and size possess a significant influence on the physical and chemical characteristics of the materials.

### 2.3. Scanning Electron Microscopy (SEM) Analysis Results

**Figure 3:**
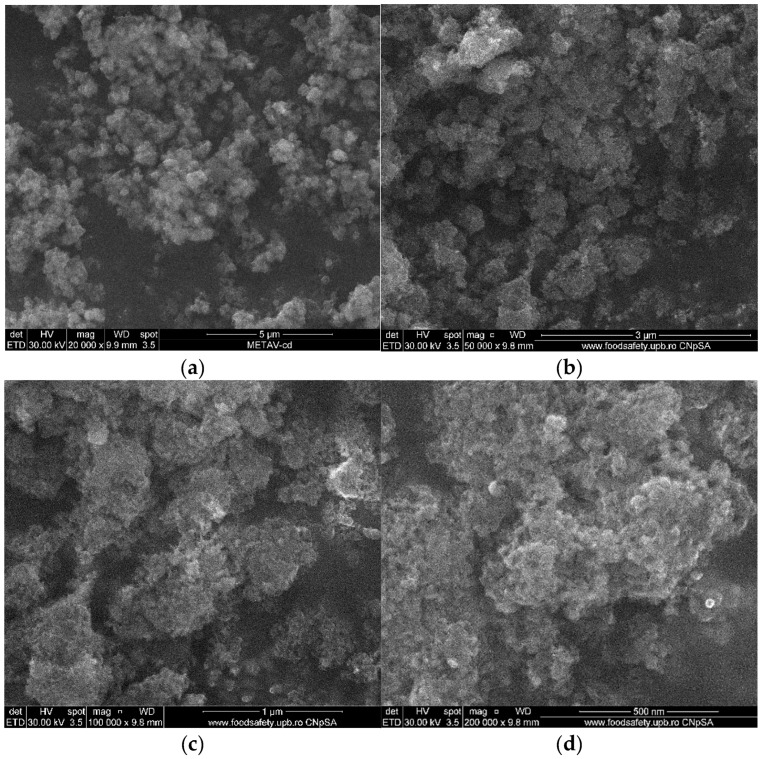
SEM images of green-synthesized untreated MgO NPs at 20,000× (**a**) and calcinated MgO NPs at (**b**) 50,000×, (**c**) 100,000×, and (**d**) 200,000× magnifications, respectively.

The shape and structure of the acquired samples are thoroughly examined in the SEM micrographs shown in Figure 3. Regarding the MgO NPs’ particle size, it was determined that they have an irregular form and range in size from 10 to 40 nm, but they also have an increased tendency to form nanoagglomerates. The main cause of this tendency is the NPs’ high surface area, which raises their surface energy and causes a natural urge to cluster. Additionally, this aggregation tendency is facilitated by the NPs’ tiny size, which ranges from 3 to 20 nm. Consequently, the NPs cluster together due to enhanced surface energy and van der Waals interactions brought on by their large surface area and small particle size [32,33].

### 2.4. Fourier-Transform Infrared (FTIR) Spectroscopy Analysis Results

**Figure 4:**
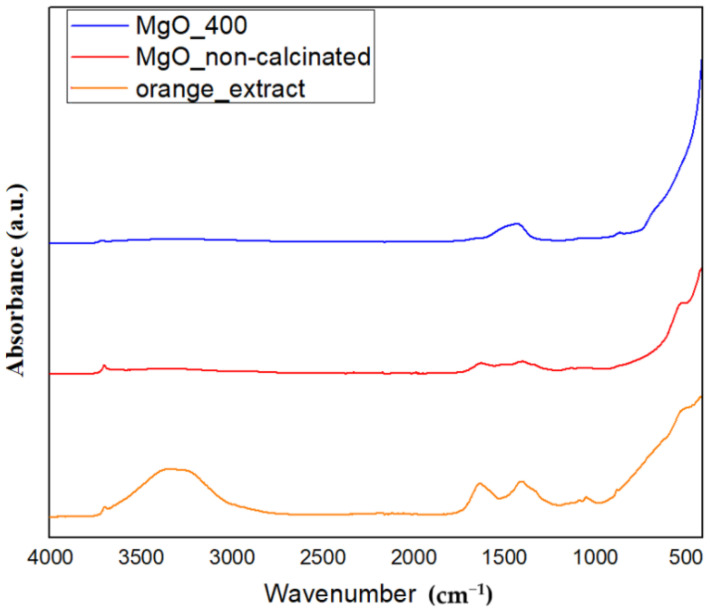
FT-IR spectra of MgO NPs, such as orange—orange extract; red—untreated MgO powder; and blue—MgO NPs calcinated at 400 °C (cm^−1^).

The FTIR spectra of the produced metal oxide NPs are shown in Figure 4. In this direction, the samples exhibit prominent absorption bands in the 400–600 cm^−1^ range (blue curves), which have been attributed to metal–oxygen (M–O) stretching vibrations and validate the synthesis of MgO NPs. These correlations are consistent with previous research on metal oxides synthesized utilizing eco-friendly processes [34].

The phytochemicals (flavonoids, terpenoids, and carboxylic acids) present in the orange peel extract, along with adsorbed water, surface hydroxyl groups, or possibly undecomposed nitrates, may also be responsible for the bands in the 1300–1600 cm^−1^ and 3400–3500 cm^−1^ regions in the non-calcined samples. However, the absence of these bands in the calcined samples supports the theory that organics and nitrates entirely decompose when heated. This conclusion is further supported by the results of TGA-FTIR evolved gas analysis, which failed to identify any NOₓ species, and XRD, which confirms the absence of nitrate-related crystalline phases in the final products. Consequently, our results clearly demonstrate that the orange peel extract does more than merely operate as a physical medium; it also plays a chemical role in the precursor transformation process.

The orange peel extract’s negative groups stabilize the extract, while its functional groups donate electrons that can react with metal ions. Therefore, it can be said that the orange peel extract’s functional groups and the metal salts interacted by donating electrons from the extract to the inorganic species, which helped them change into metallic oxide forms. Organic molecules decompose during calcination, leaving behind metal-oxide bonds and purifying the developed materials. This procedure demonstrates the manner in which natural extracts work in the synthesis of NPs by providing stabilizing agents and reactive groups for successful and environmentally friendly manufacture [35].

The transformation of the metal nitrates in the presence of orange peel extract was clearly demonstrated by TGA and FTIR of evolved gases in Section 2.1. In particular, the thermal degradation profiles revealed significant weight losses that were associated with the generation of inorganic residues and the breakdown of organic capping agents. These residues, which are far greater than what would be predicted from the decomposition of pure metal nitrate alone, suggest that there were initial interactions—likely complexation—between the metal ions and the organic molecules in the extract. Furthermore, the absence of the abrupt breakdown phases typical of unreacted metal salts in the thermal profiles suggests that the metal ions had already reacted with the extract prior to calcination.

These compounds most likely result from interactions between metal ions and the hydroxylated phytochemicals in the extract, which decompose into metal oxide NPs when heated. However, it is important to keep in mind that XRD can only identify crystalline phases. Any amorphous organic–inorganic intermediates that might exist prior to or during the early stages of calcination but are undetectable by XRD are indirectly supported by FTIR and GC-MS data, which identify organic functional groups that aid in the synthesis process. The results of TGA/FTIR, XRD, and FTIR spectroscopy provide a consistent picture of a multi-step process that involves the creation of organo-metallic intermediates and their subsequent thermally induced conversion into crystalline metal oxides. There is no proof that metal ions in our system are reduced to zero-valent metals, which is consistent with research on the environmentally friendly creation of metal oxide (not metallic) NPs.

### 2.5. GC-MS Analysis Results

The GC-MS evaluation revealed that the efficacy of green synthesis using orange peel extract is mostly due to the synergistic activity of its several bioactive components (Figure S1). The extract from orange peel contains terpenoids, polymethoxyflavones, fatty acids, and derivatives of sugar and amino acids that are bioactive by nature. According to Table 1, they interact to support morphological control, stability, and reduction in the MgO NPs. The terpenoids produce controlled reduction and chelation by acting as both electron donors and metal ion complexing agents. While fatty acids function as natural surfactants that aid in particle capping, dispersion stability, and morphological alteration, these compounds, which are related to polymethoxyflavones, have a high redox potential and exhibit dual-function activity in reduction and chelation. Furthermore, sugar derivatives and amino acids—especially those that are altered during extraction—assist in modifying the size and structure of particles.

Coordinated biochemical activity is maximized by the conditions of synthesis and extraction. These processes produce effective and consistent NPs by preserving and activating the biofunctional components. The stability and reproducibility of the produced NPs are explained by this technique.

The chemical complexity of the orange peel extract, which points to the possibility of a multi-step, coordinated mechanism of NP formation, is particularly intriguing. Unlike traditional green syntheses that rely on a single class of compounds (like phenolics or flavonoids), our GC-MS analysis revealed a rich mixture of polymethoxyflavones (like tangeretin and nobiletin), terpenoids, fatty acids, and derivatized sugars, each of which contributes independently to the reduction and stabilization process. Terpenoids and flavones reduce metal ions and promote nucleation [36], amino acid derivatives change morphology, and fatty acids aid in particle capping and colloidal stabilization [37]. Consequently, it can only be concluded that these compounds probably function in concert.

### 2.6. Zeta Potential Analysis Results

As shown in Table 2, the green-synthesized MgO NPs’ zeta potential was further evaluated. In this direction, one of the most important factors that controls how NPs interact with bacterial cell surfaces is zeta potential. The capacity of the produced NPs to act as drug delivery carriers is largely determined by its value. In order to increase the permeability of bacterial membranes and stimulate antibiotic activity, negatively charged carriers are generally favored [38]. On the other hand, via electrostatic attraction, NPs with a higher positive surface charge can enhance their contacts with fungal cells, facilitating the release of metallic ions and, thus, preventing microbial development [39].

In addition, the synthesized MgO NPs exhibit a positive zeta potential value of 11.74 ± 0.49 mV, indicating a positively charged surface. Given that metal oxide NPs were generated without the use of additional stabilizers, this intermediate result indicates low colloidal stability. Although surface charge may be influenced by residual organic moieties (FTIR, Section 2.4), the extract’s main function is precursor transformation rather than long-term stability.

It is important to point out that the zeta potential for magnesium oxide is in the moderate range in terms of magnitude. In general, moderate colloidal stability is linked to zeta potential values between −30 mV and +30 mV, whereas high stability is indicated by values above these limits. Strong electrostatic repulsion may not be ensured by the comparatively low value of 11.74 mV, which could permit nanoparticle aggregation in suspension [40].

Interestingly, because of the significant difference in electronegativity between Mg and O (2.13) compared with other metal oxides, MgO has the most noticeable ionic property among typical metal oxides. In contrast to oxides with higher covalent contributions, this increased ionic bonding lessens surface polarization effects, which could account for the lower observed zeta potential. These results demonstrate complex interactions among chemical bonding, surface characteristics of NPs, and electrokinetic activity. Furthermore, it was determined that although the orange peel extract aids in the production of MgO NP, it has no apparent impact on the long-term colloidal stability of the calcined product because the zeta potential stays in the moderate range.

### 2.7. Transmission Electron Microscopy (TEM) Analysis Results

**Figure 5:**
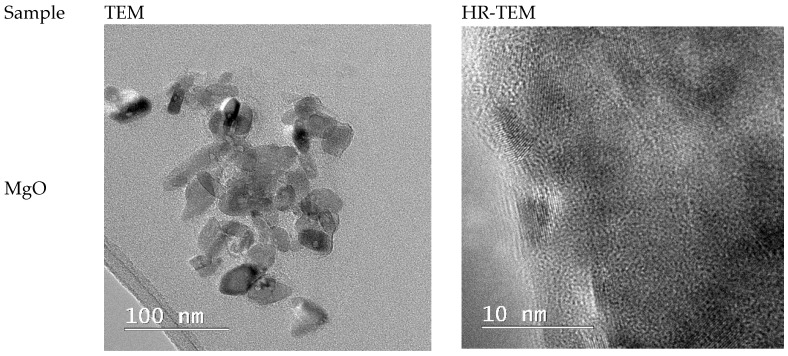
Transmission electron microscopy (TEM) images and high-resolution transmission electron microscopy (HR-TEM) images for MgO samples.

Figure 5 shows TEM images of the green-synthesized MgO NPs. SEM micrographs revealed that the MgO NPs possess uneven morphologies, with particle sizes ranging from 10 to 40 nm, and the TEM observations confirm this heterogeneity in shape and size. The tendency of these particles to form nanoagglomerates is likely due to insufficient electrostatic stabilization—consistent with the moderate zeta potential values—rather than their nanoscale dimensions alone. High-resolution TEM (HRTEM) images show that the MgO NPs are crystalline, consisting of smaller crystallites within the larger particles.

**Figure 6:**
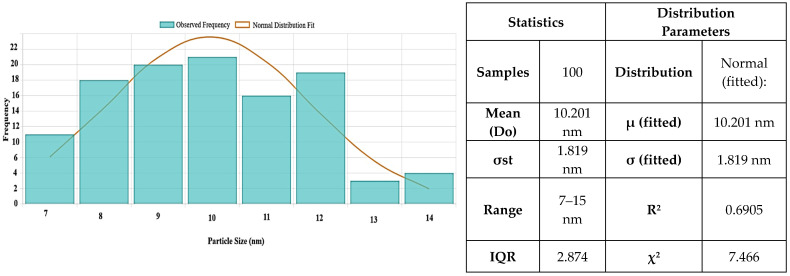
Histogram of MgO NP sizes with descriptive statistics and fitted distribution parameters. The sample (n = 100) shows a mean size of 10.201 nm, low variability (CV = 10.0%), and strong normality (R^2^ = 0.6905), indicating uniform synthesis. The results are derived exclusively from TEM micrographs and not from DLS measurements.

Figure 6 shows the particle size distribution for MgO NPs. With a larger size range of 6 to 40 nm, the histogram analysis showed a mean particle size of 10.2 ± 1.81 nm. Although the synthesis produces primarily small NPs, there is a degree of polydispersity present, as seen by the majority of particles falling between 6 and 16 nm.

Additional information on their crystallinity can be obtained by comparing the diameters of the particles as determined by SEM and TEM (Figure 3 and Figure 5) and crystallites as determined by XRD (Figure 2). The crystallite size determined by XRD (9.08 nm) is marginally smaller than the typical MgO particle size as established by TEM (10.2 ± 1.81 nm). Given the comparatively good agreement between SEM and XRD data, the discrepancy may indicate that MgO NPs are either monocrystalline or have a modest number of crystallites per particle. A statistical examination of the TEM data (n = 100) shows a mean size of 12.586 nm, moderate variability (CV = 10.0%), and strong normality (R^2^ = 0.6905), indicating a mostly uniform synthesis process, despite the observed greater range in individual measurements. These findings show that MgO NPs produced through green synthesis have a very narrow size distribution and have high crystallinity, making them suitable for potential uses in biological systems. Additionally, they support the morphological information from SEM and TEM.

### 2.8. Antioxidant Capacity of MgO Nanoparticles

**Figure 7:**
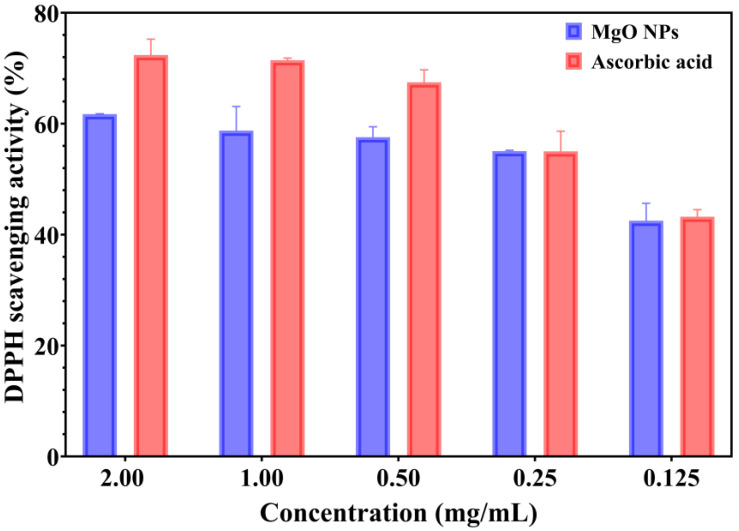
Antioxidant activity of MgO NPs using the DPPH assay.

The antioxidant properties of green-synthesized MgO NPs were assessed using a spectrophotometric method, which is based on the ability of samples to reduce the DPPH reagent. The color changes from purple to yellow indicate the strong antioxidant properties, and the ability to neutralize the reactive oxygen species (ROS) [41]. The antioxidant activity of MgO NPs compared with a standard/positive control (ascorbic acid) is presented in Figure 7.

The capacity of green-synthesized MgO NPs to scavenge the DPPH radical is a dose-dependent response. Moreover, the MgO NPs showed DPPH inhibition from 42.50 to 61.73%. Notably, the values for the standard control (ascorbic acid) are appropriate to tested NPs, especially for lower concentrations (0.25 and 0.125 mg/mL). The DPPH scavenging activity of ascorbic acid ranged from 43.23% to 72.38% (for 0.125–2.00 mg/mL concentrations). Otherwise, the data results suggest that green-developed MgO NPs have significant antioxidant properties; however, their properties are not as effective as those of ascorbic acid.

### 2.9. Cell Viability Assessment Results

**Figure 8:**
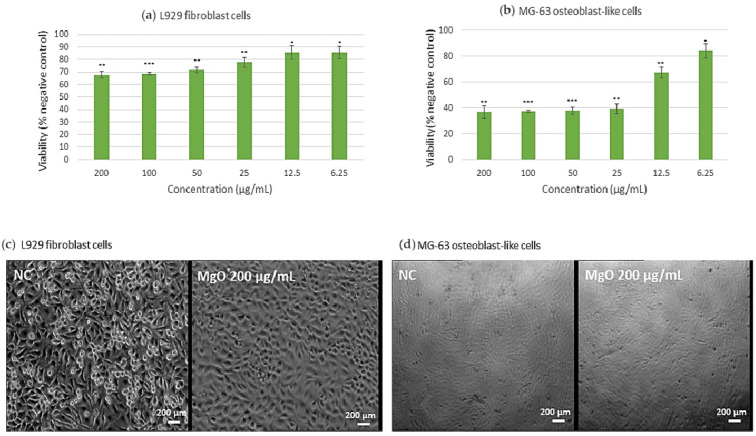
Cytotoxicity assessment of green-synthesized MgO NPs after 4 days of incubation for (**a**,**c**) murine fibroblast L929 cells and (**b**,**d**) MG-63 osteoblast-like cells. The negative control consisted of cells incubated only with complete culture medium. Data are expressed as mean ± SD. Statistical analysis was performed using Student’s *t*-test (* *p* < 0.05, ** *p* ≤ 0.01, *** *p* ≤ 0.001).

To investigate the cytotoxic effects across multiple cell lines, an MTT assay was performed, and presented in Figure 8. Thus, as shown in Figure 8, MgO NPs displayed good biocompatibility for L929 murine fibroblast cells (Figure 8a), with cellular survival above 80% at all tested doses, satisfying ISO 10993-5 [42] biocompatibility requirements. This high level of biocompatibility implies low cellular stress and stable metabolic activity. In MG-63 osteoblast-like cells (Figure 8b), a more pronounced response was observed: cellular metabolism exhibited an inverse association with NP concentration up to a limit of 25 µg/mL, followed by a linear response pattern at higher levels. Significantly, the comprehensive morphological study demonstrated that, regardless of the observed changes in metabolic activity, both cellular shape and population density were consistent with the negative control. This pattern suggests that MgO NPs preserve the structural integrity and viability of cells while primarily influencing their metabolic processes. Instead of universal cytotoxicity, the preservation of cellular architecture in the face of metabolic regulation points to a potential particular interaction mechanism between MgO NPs and cellular metabolic pathways.

**Figure 9:**
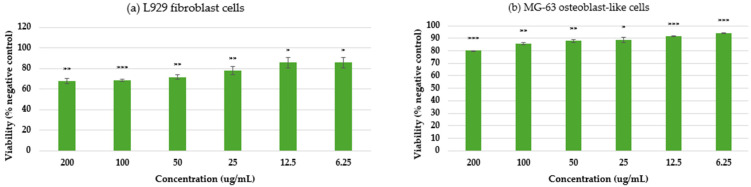
Cytotoxicity assessment of green-synthesized MgO NPs after 7 days of incubation for (**a**) murine fibroblast L929 cells and (**b**) MG-63 osteoblast-like cells. The negative control consisted of cells incubated only with complete culture medium. Data are expressed as mean ± SD. Statistical analysis was performed using Student’s *t*-test (* *p* < 0.05, ** *p* ≤ 0.01, *** *p* ≤ 0.001).

To further validate these findings, cytotoxicity was also evaluated after prolonged exposure (7 days), as presented in Figure 9. The results confirmed the high cytocompatibility of MgO NPs with L929 fibroblast cells, which maintained cell viability above 80% even at the highest tested concentrations, in line with ISO 10993-5 requirements. For MG-63 osteoblast-like cells, a more pronounced concentration-dependent effect was observed after extended incubation, with significant reductions in metabolic activity at 100 µg/mL (0.1 mg/mL). These results indicate that longer exposure amplifies the cellular response, particularly in osteoblast-like cells, while fibroblasts remain more resilient. Such outcomes highlight both the importance of exposure time and cell type specificity when assessing nanoparticle safety and biocompatibility.

### 2.10. Qualitative Evaluation of Antimicrobial Activity Assessment

**Figure 10:**
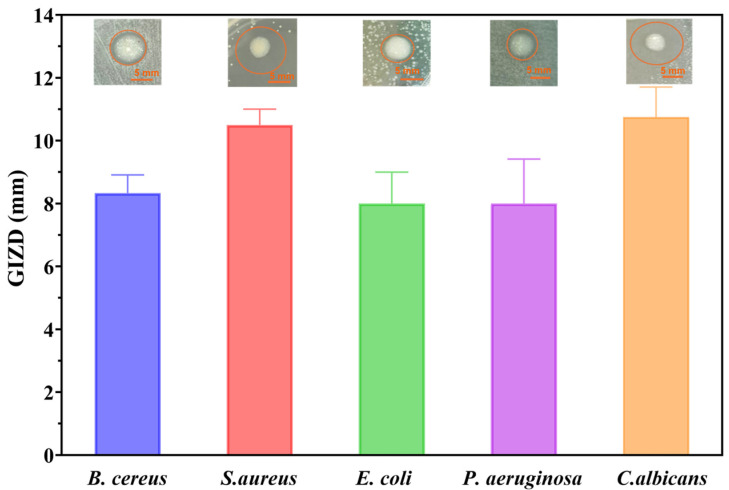
GIZD values of metal oxide NPs.

First, the growth inhibition zone diameters (GIZDs) that developed close to the spot were measured and expressed as mean values ± SD in order to qualitatively assess the antimicrobial qualities of metal oxide NPs (Figure 10).

With the obtained GIZD values, MgO NPs most significantly showed the strongest antimicrobial action against *C. albicans*. The improved interaction between the positively charged MgO surface (+11.74 mV zeta potential) and the components of the fungal cell wall is responsible for this increased antifungal activity [43].

With GIZD values of 10–11 mm, *S. aureus* and *B. cereus* also showed significant susceptibility, which is in line with the anticipated improved interaction between positively charged NPs and the thick peptidoglycan layer of *Gram-positive bacteria* [44]. Gram-negative bacteria, on the other hand, showed more varied responses. To illustrate the innate resistance mechanisms of this pathogen, *P. aeruginosa* showed moderate activity with GIZD values ranging from 6–9 mm, while *E. coli* showed lower susceptibility with GIZD values of 7–9 mm.

### 2.11. Quantitative Evaluation of Antimicrobial Activity Assessment

**Figure 11:**
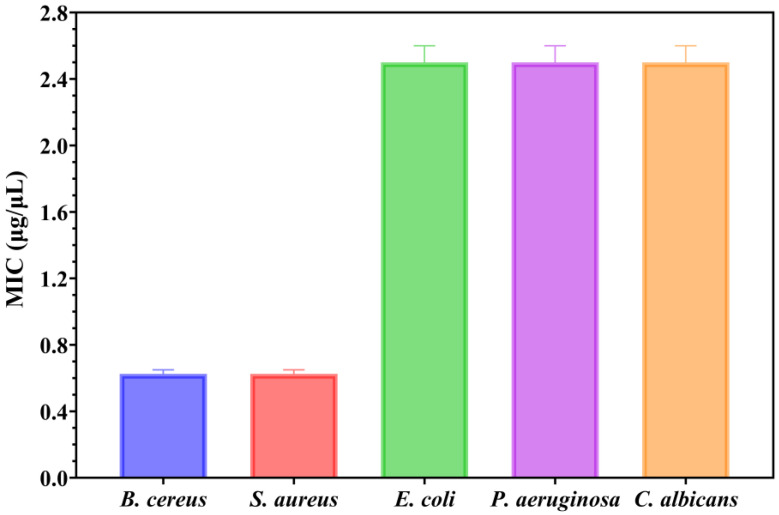
MIC values of green-synthesized MgO NPs against tested microbial strains.

Secondly, the antimicrobial assessments were continued with quantitative evaluation by determining the minimum inhibitory concentration (MIC) values (Figure 11).

Understanding the efficiency of these NPs starts from understanding that lower MIC values imply more antimicrobial potency because they represent the smallest amount of material required to suppress bacterial growth. Notably, the MIC assessment supported the previously obtained qualitative results by quantitatively demonstrating the antimicrobial properties of the NPs, as shown in Figure 11. The MIC results revealed that MgO NPs displayed the strongest antimicrobial activity against *Gram-positive bacteria*. Notably, both *B. cereus* and *S. aureus* exhibited similar sensitivity profiles, with MIC values between 0.600 and 0.650 μg/μL (mean MIC: 0.625 ± 0.025 μg/μL for each strain). Stronger electrostatic interactions between the thick peptidoglycan layer found in *Gram-positive bacteria*’s cell walls and the positively charged MgO NPs surface are responsible for this increased efficacy against these bacteria [44]. On the other hand, the fungal strain and the tested Gram-negative bacteria showed noticeably greater resistance, necessitating much higher dosages to suppress development. The MIC values for *E. coli*, *P. aeruginosa*, and *C. albicans* were almost the same, consistently reading between 2.4 and 2.6 μg/μL in all replicates (mean: 2.5 ± 0.1 μg/μL for all three strains). This pattern shows that even while the qualitative GIZD assay showed significant antifungal activity, the quantitative MIC analysis shows that *C. albicans* needs amounts comparable to Gram-negative bacteria to completely inhibit growth.

The selective antibacterial action of MgO NPs is highlighted by the almost four-fold difference in MIC values between *Gram-positive bacteria* (0.625 μg/μL) and the other examined pathogens (2.5 μg/μL). In contrast to the lipopolysaccharide outer membrane of Gram-negative bacteria or the intricate cell wall structure of fungi, the exposed peptidoglycan in *Gram-positive bacteria* offers more accessible binding sites for the positively charged NPs, which is probably the cause of this selectivity. The enhanced inhibitory effects against these more resistant strains may also be linked to the distinctive cubic morphology of MgO NPs, which could promote more efficient interactions with microbial cell walls, as supported by previous studies [24,44].

### 2.12. Semiquantitative Assessment of Microbial Adherence to the Inert Substratum Assessment

**Figure 12:**
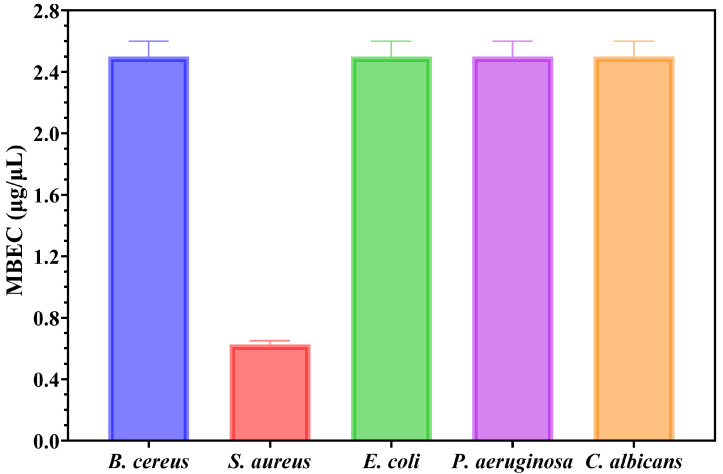
MBEC values of green-synthesized MgO NPs against tested microbial strains.

The minimal biofilm eradication concentration (MBEC) assessment gives critical information about how well these NPs can inhibit the adherence of biofilms. The antimicrobial effects of MgO NPs were evaluated by assessing their effect on pathogenic strain adhesion to an inert substrate. The MBEC values are illustrated in Figure 12 and confirm the qualitative and quantitative results (Figure 10 and Figure 11).

The results reveal distinct differences in susceptibility among the tested strains. *S. aureus* biofilms showed remarkable sensitivity to MgO NPs. In this direction, total eradication had been achieved around 0.625 μg/μL, which was the lowest MBEC value of any examined strains. This increased antibiofilm activity supports the unique efficacy of MgO NPs against this Gram-positive strain and is consistent with MIC values.

For example, *B. cereus*, *E. coli*, *P. aeruginosa*, and *C. albicans* all needed about 2.5 μg/μL to completely eradicate biofilms, which is four times more than what *S. aureus* requires. It is noteworthy that *B. cereus*, although Gram-positive like *S. aureus*, needed the same higher dose as the fungal and Gram-negative strains, indicating that susceptibility is influenced by biofilm architecture more so than cell wall structure. With a particularly strong activity against *S. aureus* biofilms, the MBEC results demonstrate the broad-spectrum antibiofilm potential of MgO NPs. This makes these NPs a promising option for applications that need antibiofilm properties against persistent, treatment-resistant biofilm formers.

## 3. Discussion

Our study focuses solely on the synthesis of MgO NPs using orange peel extract under controlled experimental settings, even though there is a wealth of evidence supporting the eco-friendly manufacture of metal oxide NPs using plant extracts. Comprehensive physicochemical analysis (TGA/DSC, XRD, FTIR, GC-MS, SEM/TEM, and zeta potential studies) suggests that the phytochemicals in orange peel extract may have a variety of functions, including reducing, chelating, stabilizing, and morphology-directing agents during NP formation. These bioactive substances actively interact with precursor salts, according to thermal analysis, indicating chemical changes that go beyond straightforward nitrate breakdown. Most organic residues were successfully eliminated by calcination, producing extremely crystalline MgO phases. Through a variety of processes, the phytochemicals found in orange peel extract make a substantial contribution to the synthesis process. Biomolecules derived from plant extracts have been shown by Abinaya et al. [45] to serve as capping agents on the surfaces of MgO particles, affecting their shape and biological activity. By methodically optimizing extraction and precursor concentrations, our synthesis yielded MgO NPs with a narrow size distribution (~9 nm crystallite size), which contrasts favorably with reported particle sizes in the literature that range from 29.5 to 85.8 nm.

Furthermore, the TGA analysis showed that the initial weight loss observed below 200 °C in all samples is mostly due to the removal of adsorbed moisture from the particle surfaces [46]. The degradation of the capping phytochemicals present in the uncured samples is responsible for the second notable weight loss, which occurs between 200 and 400 °C. The organic residues’ oxidation and decomposition are represented by the exothermic and endothermic peaks in this region, respectively. Furthermore, the theoretical value of the metallic nitrates is substantially lower than the residual mass of all samples. We may establish that the metal nitrates were actually changed during the reaction with the orange peel extract because phytochemicals are also present in all samples. Furthermore, the existence of these distinct thermal processes indicates the efficacy of green synthesis since they validate the progressive oxidation of metal precursors and their full transformation into oxide forms at elevated temperatures [47]. This phase evolution is critical for producing stable, highly crystalline NPs appropriate for biological applications.

The cubic crystalline phase of MgO, with an average crystallite size of about 9 nm, was verified by XRD assessment. The effectiveness of our extract-mediated method is demonstrated by the fact that this value is lower than those found in previous green syntheses (e.g., Pallavi et al. [48] and Al-Harbi et al. [49]). All samples displayed a moderate level of agglomeration, which is characteristic of high-surface-energy NPs, and SEM pictures showed irregularly shaped particles, which were in agreement with TEM observations. Every sample exhibited an agglomeration tendency, which is less noticeable than that observed in chemical synthesis methods but typical of high-surface-energy NPs. The FTIR and GC-MS results offer indirect but compelling evidence for a possibly distinct nanoparticle formation mechanism in our system. Orange peel extract’s diverse and complementary chemical constituents, including fatty acids, terpenoids, polymethoxyflavones, and derivatized sugars, appear to interact. Most likely, our synthesis mechanism has been made up of multiple cooperating activities. First, by serving as electron donors, flavonoids and terpenoids lower metal ions. Additionally, their chelating properties help to stabilize the newly formed metal nuclei. While sugars and amino acids seem to influence particle shape during crystal formation, fatty acids cap the surfaces of the NPs to prevent them from aggregating. This multi-pathway strategy is a major improvement over conventional green synthesis methods, which often rely on single reduction mechanisms.

The rich phytochemical composition of orange peel extract, with its numerous functional groups, creates a unique environment that improves stability, crystallinity, and attains the right size uniformity. Our results clearly demonstrate the advantages of this multi-component, environmentally friendly method for producing NPs, even though more mechanistic studies (such in situ spectroscopy) would provide definitive proof. This discovery opens up new possibilities for developing more efficient and environmentally friendly synthesis methods that are inspired by the complexity of nature. The positively charged surface of all NPs is shown by their positive zeta potential values, which improve electrostatic interaction with negatively charged bacterial cell membranes and increase antibiotic activity [50]. Since aggregation may reduce the NPs’ efficacy in biological applications, the zeta potential degree suggests moderate colloidal stability, highlighting the possibility that stabilizers may be necessary for prolonged storage or usage under specific circumstances. Additionally, TEM analysis demonstrated the NPs’ crystalline nature and demonstrated that MgO samples have distinct crystal shapes. SEM results are consistent with the irregular shape of MgO NPs shown in TEM images. These NPs were created during green synthesis when the orange peel extract causes reduction and stabilization, and high-resolution TEM scans showed that they are extremely crystalline with distinct crystal shapes. These NPs are primarily monocrystalline, which means that each particle is made up of a single crystal domain rather than several crystallites, as confirmed by the strong agreement between the particle sizes determined by TEM/SEM and the crystallite sizes determined by XRD. Since stability is known to impact cell uptake and functionality, these characteristics are crucial for application in biological contexts. Given that particle size and shape might affect cell interaction in medical applications, the green synthesis process appears to offer a dependable and repeatable means of producing NPs with a high degree of structural homogeneity.

Our study of the green-synthesized NPs showed unique patterns of cellular interaction and biocompatibility in the context of nanoparticle-based medicinal methods. In line with Pallavi et al. [48], MgO NPs preserved >80% viability in fibroblasts at 25 mg/mL; however, their size distribution was more constrained (9 nm compared to 15 nm in their work), most likely as a result of our extract-mediated synthesis. Furthermore, at 25 µg/mL, our MgO NPs preserved >80% fibroblast vitality, while chemically generated MgO NPs needed 50 µg/mL to attain comparable viability, according of Al-Harbi et al. [49]. Therefore, without influencing cellular viability or density, the MgO NPs produced in this study preserve cellular viability in fibroblast cells by more than 80% (fulfilling ISO 10993-5 requirements) while exhibiting an intriguing metabolic modulator regulating influence on MG-63 osteoblast-like cells. These beneficial characteristics make MgO NPs an excellent option for a range of tissue engineering applications. Furthermore, their strong affinity for fibroblasts points to a great deal of promise for applications in wound healing, where tissue regeneration depends on sustained cell viability. The unique characteristics of every form of nanoparticle indicate several ideal use cases when considering practical uses. Additionally, Go et al. [51] have stated that the distinct metabolic modulation shown with MgO NPs may be especially beneficial in bone tissue engineering applications, where appropriate tissue creation may be supported by controlled cell metabolism. Pinho et al. [52] have also provided an explanation for similar findings.

MgO NPs’ unique physicochemical characteristics, such as their crystalline structure, surface charge, and nanoscale size, are directly linked to their antimicrobial activity. All studied pathogens showed moderate but constant antimicrobial action from our green-synthesized MgO NPs, with many complementary mechanisms largely controlling their efficacy. According to Sirelkhatim et al. [53], the main mechanism is electrostatic interactions between negatively charged microbial cell membranes and the positively charged nanoparticle surface (+11.74 mV zeta potential), which break the membrane and cause cell death. Furthermore, when MgO NPs encounter microbial cells, they can produce reactive oxygen species (ROS), which can lead to oxidative stress and harm to cellular constituents such proteins, lipids, and DNA. Through the disruption of vital cellular and enzyme functions, the release of Mg^2+^ ions from the NPs may also contribute to antibacterial activity. According to the qualitative evaluation, MgO NPs were most effective against *S. aureus* and *Candida albicans*, but Gram-negative bacteria responded in inconsistent ways.

*Gram-positive bacteria* (*B. cereus* and *S. aureus*) had similar sensitivity profiles, while *E. coli*, *P. aeruginosa*, and *C. albicans* needed significantly higher concentrations, indicating a four-fold difference in antimicrobial potency, according to the quantitative MIC assessment.

Additionally, the antibiofilm assessment (MBEC) identified significant clinical implications. The biofilms of *S. aureus* showed remarkable sensitivity, eradicating the biofilm completely at 0.625 μg/μL, whereas the other bacteria examined needed 2.5 μg/μL to do the same. The development of extracellular polymeric substances and biofilm architecture is an important factor in determining antimicrobial sensitivity, according to this observation. Our MgO NPs’ cubic structure may allow for efficient penetration of EPS matrices, as suggested by Rotti et al. [24] and validated by Sadeghzadeh et al. [54]. MgO NPs’ selective antibacterial effect is demonstrated by the four-fold difference in MIC values between *Gram-positive bacteria* and other pathogens, which makes these green-synthesized MgO NPs attractive options for antimicrobial applications.

Although preliminary measurements of ROS generation by these NPs were not carried out in this study, prior research has demonstrated ROS formation by similar metal oxide NPs under similar synthesis and environmental conditions [3,55,56], which provides strong support for the antimicrobial efficacy of the synthesized MgO NPs.

For instance, the team under the direction of Ahamed et al. [57] generated magnesium oxide nanoparticles (MgO NPs) using garlic extract and showed notable antibacterial and anticancer properties, which was partially because the reactive oxygen species they produced caused oxidative stress in the target cells. The significance of ROS in their toxicity-activity balance has also been highlighted by Al-Harbi et al. [49], who showed that green-synthetized MgO NPs can produce ROS such as hydrogen peroxide, which causes membrane damage and bacterial cell death. In addition, Girma et al. [58] demonstrated that their bio-synthesized MgO nanoparticles exhibited significant biological activity, particularly strong antioxidant properties. Using DPPH assays, they showed the MgO NPs achieved excellent free radical scavenging activity, ranging between 79.8% and 93.9%, which is comparable to the standard antioxidant ascorbic acid. In addition, the nanoparticles also displayed effective reactive oxygen species (ROS) scavenging activity (75.5–89.3%), indicating their potential as powerful antioxidant agents for biomedical applications. Similarly, Amor et al. [59] emphasized that smaller particle size (4.6 nm for shrimp shell chitosan-derived MgO) and higher crystallinity contribute to better antioxidant performance. The MgO NPs showed potential as effective antioxidants due to their ability to scavenge free radicals, indicating their promise for biomedical applications. The group Ali et al. [60] reported the green synthesis of MgO NPs using *Magnolia champaca* plant root extract and evaluated their biological activities. The MgO NPs showed strong antioxidant activity assessed by the DPPH assay, with a dose-dependent radical scavenging effect reaching up to 68.9% at 120 μg/mL concentration, which was higher than both the plant extract alone and the ascorbic acid standard at the same concentrations. Furthermore, by using plant extracts, Rashid et al. [61] provided a summary of several green biosynthesis techniques and highlighted the antibacterial and photocatalytic qualities of the generated MgO NPs that are connected to the production of ROS. The formation of ROS by MgO NPs synthesized by environmentally friendly methods is a critical mechanism for lipid peroxidation and microbiological growth inhibition, according to another study conducted by Rotti et al. [24].

This conclusion highlights the significance of ROS in toxicity-activity trade-offs by showing that MgO NPs, which have shown lesser ROS production in previous studies [3], demonstrated moderate antibacterial activity but higher biocompatibility. Future research will use direct ROS detection techniques like electron spin resonance spectroscopy or fluorescent probe assays (like DCFH-DA) to measure ROS production by our green-synthesized NPs in order to conclusively prove these pathways. Furthermore, ROS may contribute to the antimicrobial activity of MgO NPs, according to our results; however, this conclusion is based on previous studies of comparable nanostructures rather than direct observations.

Additionally, a detailed characterization of MgO NPs revealed a number of significant structure–property relationships that explain their diverse biological performances. Cell viability was highest in the small crystallites (MgO~9 nm) (>80% at all concentrations). This suggests that there is a direct relationship between biocompatibility and crystallite size as assessed by XRD analysis. Smaller particles with a higher surface area-to-volume ratio may be the cause of this impact since they encourage deeper cell-to-cell contacts. Additionally, the crystal structure had a significant impact on antibacterial activity. Although other metal oxide NPs have stronger action against planktonic bacteria, the irregular form of MgO NPs (10–40 nm) appears to allow better penetration of *P. aeruginosa* biofilms.

Important directions for customizing NPs for biomedical applications are provided by these correlations between structure and property. In the meantime, further research needs to be carried out on their possible uses in a wider variety of tissue engineering and wound healing applications. This thorough knowledge of the interactions between NPs and cells offers important insights into creating next-generation biomaterials, which will ultimately lead to more successful tissue engineering and wound-healing techniques. Furthermore, our results highlight the importance of carefully considering both concentration and cell-type-specific reactions when developing therapeutic strategies based on NPs.

Therefore, the systematic utilization of the intricate phytochemical profile of orange peel extract for regulated MgO NP production serves as what makes this work innovative. Our method uses the coordinated action of several bioactive classes, including terpenoids for reduction and chelation, polymethoxyflavones for redox control, fatty acids for surface capping, and derivatized sugars for morphological guidance, to achieve unprecedented control over nanoparticle characteristics, in contrast to conventional green synthesis approaches that rely on single reducing agents. Highly crystalline MgO NPs with a narrow size distribution (9 nm crystallites) and a distinct biological profile that combines remarkable biocompatibility (>80% viability) with selective antibacterial activity are produced by this multi-pathway production process. Furthermore, a rational foundation for creating green-synthesized metal oxide NPs with specific biological properties is provided by the definitive characterization of structure–activity relationships.

## 4. Materials and Methods

### 4.1. Materials

Magnesium nitrate hexahydrate [Mg(NO_3_)_2_·6H_2_O, ≥98%, Fluka, Buchs, Switzerland] has been selected as a reagent for the synthesis of MgO NPs. Ammonia solution (25%, Merck, Darmstadt, Germany) was used for pH adjustment in MgO synthesis. Two kilograms of oranges were bought from a neighborhood market in order to make the orange peel extract. 2,2-diphenyl-1-picrylhydrazyl (DPPH), ascorbic acid and ethanol from Sigma-Aldrich (Darmstadt, Germany) were used for antioxidant activity of synthetized NPs. Immortalized murine fibroblast L929 cells, primary human dermal fibroblast BJ cells (both from the American Type Culture Collection, ATCC, Manassas, VA, USA), and MG-63 osteoblast-like cells (Cell Lines Service GmbH, Heidelberg, Germany) were cultured in Dulbecco’s Modified Eagle Medium (DMEM) supplemented with 10% fetal bovine serum (FBS) and 1% Penicillin–Streptomycin antibiotic solution to assess cell viability and prepare cell cultures. Cells were cultivated at 37 °C with 90% relative humidity in a humidified environment with 5% CO_2_, following conventional physiological conditions. Using supplies from Sigma-Aldrich, the microbiological activity was carried out using Nutrient Broth No. 2, Sabouraud, agar, phosphate buffer saline, methanol, crystal violet (purple), and acetic acid. The University of Bucharest’s Department of Microbiology, Faculty of Biology & Research Institute, supplied all of the strains used in this investigation.

### 4.2. Preparation of Orange Peel Extract

Fresh oranges weighing close to 2 kg were bought from a neighborhood market. After being cleaned and chopped, the peels were dried for four hours at 40 °C in an oven. The dried peels were ground into a fine powder in a mortar for 30 min. The extract was made by adding 2 g of peel powder (1% *w*/*v*) to 200 milliliters of deionized water and stirring it magnetically for one hour at room temperature. Ultrasonic treatment for one hour at 60 °C was performed after this procedure. For future utilization, the mixture was stored at 4 °C after being filtered through Whatman No. 1 filter paper. The pH of the resulting orange peel extract was approximately 5.2, consistent with the presence of organic acids such as citric, malic, and ascorbic acid [62,63].

### 4.3. Green Synthesis of Nanoparticles

Orange peel extract was used as a stabilizing and reducing agent in the green synthesis process used to create MgO NPs. A 0.2 M aqueous solution of Mg(NO_3_)_2_·6H_2_O (50 mL) was mixed with 50 mL of orange peel extract at a 1:1 volume ratio. Under constant stirring, the extract was added to the metal salt solution drop by drop. A 25% ammonia solution was used to get the pH to 12 in order to guarantee that magnesium hydroxide would precipitate completely. To ensure a good reaction and the creation of NPs, the mixture was agitated for four hours at room temperature. Following stirring, the product was filtered, dried for 12 h at 150 °C, and then calcined for 2 h at 400 °C at a rate of 5 °C per minute. In order to prevent moisture absorption until further characterization, the final MgO NP powder was kept in an airtight container under desiccation.

### 4.4. Thermogravimetric Analysis

Using a Netzsch TG 449C STA Jupiter instrument (Netzsch, Selb, Germany), the thermal behavior of dried powder precursors of MgO NPs was examined in order to determine the ideal calcination temperature and investigate any potential modification of the metallic nitrates brought on by the orange peel extract. In an alumina crucible, the powder samples were heated from ambient temperature to 900 °C at a rate of 10 °C per minute while being circulated with 50 mL of dried air per minute. A Bruker FTIR Tensor 27 (Bruker Co., Ettlingen, Germany) with an internal thermostatic gas cell was used to study the developed gases while they were being transferred via heated transfer lines.

### 4.5. X-Ray Diffraction (XRD) Analysis

A PANalytical Empyrean device (Malvern Panalytical, Cedar Park, TX, USA) operating at 45 kV and 40 mA in Bragg–Brentanno geometry with CuKα radiation (λ = 1.5418 Å) was used to perform the X-ray diffraction investigation. In addition to a 0.02 mm Ni filter installed on a PIXCel3D detector (Malvern Panalytical, Cedar Park, TX, USA) on the diffracted beam side, the diffractometer had a 0.02° Soller slit, a 1/4° fixed divergent slit, and a 1/2° anti-scatter slit on the incident beam side. A scan range of 10.0000 to 80.0107° 2θ, a step size of 0.0263°, and a counting duration of 255 s per step were among the measurement characteristics. The ICDD PDF4+ 2022 database and HighScorePlus software version 3.0.e were used to carry out data reduction, search, and match processes.

### 4.6. Scanning Electron Microscopy (SEM) Analysis

To determine the size and morphology of the green-synthesized NPs, Scanning Electron Microscopy (SEM) was performed. Images were captured using a Quanta Inspect F50 (ThermoFisher Scientific, Hillsboro, OR, USA) scanning electron microscope, which was equipped with a field emission gun electron (FEG) providing a resolution of 1.2 nm.

### 4.7. Fourier-Transform Infrared (FTIR) Spectroscopy Analysis

A Nicolet iS50 FTIR spectrometer (Nicolet, MA, USA) with a DTGS detector that offers high sensitivity in the range of 4000 cm^−1^ to 400 cm^−1^ and a resolution of 4 cm^−1^ by averaging 32 scans to improve spectral quality was used to detect the presence of functional groups within the synthesized NPs. Room temperature was used for the measurements. Omnic32 was used for data recording and processing.

### 4.8. GC-MS Analysis

A Thermo Scientific TRACE 1310 gas chromatograph, a TSQ-8000EV0 triple quadrupole mass spectrometer (Thermo Fisher Scientific, Waltham, MA, USA), and a TriPlus RSH autosampler (Thermo Fisher Scientific, Waltham, MA, USA) were used to determine the chemical constituent profile of the extracted orange peel. A Zebron ZB-5MS capillary column (30 m × 0.25 mm ID × 0.25 µm film thickness, Phenomenex, Torrance, CA, USA) was used to extract volatile chemicals. High-purity helium (99.999%) was used as the carrier gas, and the flow rate was maintained at 1.0 mL/min. Data collection ranged from 40 to 650 *m*/*z* at 0.2 s for each scan using the electron ionization (EI) mode at 70 eV. After maintaining the injector at 280 °C, 1 µL of sample was added with a split ratio of 10:1.

The oven temperature program started at 60 °C (maintained for 1 min), increased by 10 °C/min to 190 °C, then increased by 15 °C/min to the final working temperature of 300 °C, which was maintained for 30 min. Temperatures of 280 °C and 230 °C were chosen for the transfer line and ion source, respectively.

A total of 72 mL of orange peel extract was extracted twice using 35 mL parts of hexane in order to separate non-polar chemicals. Anhydrous sodium sulfate was used to dry the mixed organic layer, and a rotary evaporator (Büchi R-300, 40 °C, 300 mbar, BÜCHI Labortechnik AG, Flawil, Switzerland) was used to condense it to around 1 mL. Before the GC-MS injection, the dried residue was redissolved in 1 mL of HPLC-grade ethyl acetate after the remaining solvent was completely removed with a mild nitrogen stream. A total of 0.5 mL of the aqueous phase was dried under nitrogen and derivatized using 100 μL of BSTFA with 1% TMCS at 70 °C for 30 min in order to analyze the polar components. Data collection and analysis were conducted using Chromeleon 7.3 software. Retention indices were used for additional confirmation when needed, and mass spectra were compared to records in the NIST library to identify the compound. Peak regions in total ion chromatograms were used to semi-quantify the major components, which include terpenoids and methoxyflavones.

### 4.9. Zeta Potential Analysis

Zeta potential was measured using a Beckman Coulter (Brea, CA, USA) DelsaMax Pro instrument with a 532 nm laser. In order to prepare the samples, powder samples were sonicated for 10 min in ultrapure water to create suspensions, which were then injected into the measurement cell of the apparatus. For every measurement, six individual acquisitions were taken and recorded.

### 4.10. Transmission Electron Microscopy (TEM) Analysis

ThermoFisher Scientific’s high-resolution Titan Themis equipment (Hillsboro, OR, USA) was used for the transmission electron microscopy (TEM) assessment. A small portion of each powder sample was dissolved in deionized water using ultrasonic treatment for ten minutes prior to analysis. A 400-mesh lacey carbon-coated copper grid was then covered with 10 μL of the solution, which was then left to dry at room temperature. The accelerating voltage used to operate the microscope was 200 kV.

### 4.11. Antioxidant Activity

Determination of free radical scavenging capacity of MgO NPs was performed by using 2,2-diphenyl-1-picrylhydrazyl (DPPH). This assessment is a simple method for evaluating antioxidant capacity due to the stability of DPPH [58,64]. Initially, 3.5 mg of DPPH was dissolved in 15 mL ethanol and vortexed until solubilization. Different concentrations of MgO NPs (0.125–2 mg/mL) were prepared in ultrapure water, and 20 µL of NPs suspension and 180 µL DPPH solution were added to microcentrifuge tubes. The samples were incubated for 30 min in the dark, and spectrophotometric measurements were performed at 517 nm. Likewise, the positive control solution (ascorbic acid) was prepared. The percentage of DPPH inhibition was calculated using Equation (1).

(1) $$DPPH\;Inhibition\left(\%\right)=\left(\frac{A-B}{A}\right)\times 100$$

A represents the absorbance of the oxidized solution in the absence of antioxidant agents (control—DPPH) and B is the absorbance of the oxidized solution in the presence of antioxidant agents (MgO or ascorbic acid).

### 4.12. Biological Analysis

#### 4.12.1. Cell Culture Preparation

MG-63 osteoblast-like cells (Cytone, Heidelberg, Germany) and L929 fibroblast cells (ATCC, Manassas, VA, USA) were cultured in Dulbecco’s Modified Eagle Medium (DMEM) supplemented with 10% fetal bovine serum and 1% Penicillin–Streptomycin, in standard conditions of temperature and humidity (37 °C, 5% CO_2_, 90% humidity).

For experimental procedures, cells were seeded at a density of 5 × 10^4^ cells/mL (equivalent to 5000 cells in 100 µL per well) in 96-well tissue culture plates. The plates were then incubated under standard physiological conditions for 24 h, to facilitate cellular adhesion. Following the initial incubation period, the culture medium was removed and replaced with a fresh medium containing various concentrations of NPs, ranging from 6.25 to 200 µg/mL, prepared through serial binary dilutions. The treated cells were subsequently incubated under standard physiological conditions for 4 days, after which cell viability assessments were performed to evaluate the biological response to nanoparticle exposure. All experiments were conducted by standard cell culture protocols, and appropriate controls were maintained throughout the study period.

#### 4.12.2. Cell Viability Assessment

Cell viability assessments were conducted using the MTT (3-(4,5-dimethylthiazol-2-yl)-2,5-diphenyltetrazolium bromide) colorimetric assay. The cell monolayer was exposed to an MTT solution in a complete culture medium after the supernatant was collected. This solution was made by diluting the MTT stock solution (5 mg/mL in PBS) to a final concentration of 0.5 mg/mL in complete DMEM. Following that, the cells were cultured for two hours at 37 °C with 5% CO_2_ under standard physiological conditions. The fundamental concept behind this technique is that mitochondrial dehydrogenases seen in metabolically active cells enzymatically reduce the tetrazolium salt to formazan crystals. The quantity of live cells directly correlates with the degree of intensity of this conversion.

The medium containing MTT was removed after the appointed incubation period, and the formazan precipitate was in DMSO. UV-VIS spectrophotometry was used to measure the optical density at λ = 570 nm in order to carry out Formazan quantification. The percentage of treated samples’ absorbance compared to the negative control (considered 100% vitality) was used to express relative cell viability.

#### 4.12.3. Qualitative Evaluation of Antimicrobial Activity

The qualitative antimicrobial activity was performed using an adapted spot diffusion method, according to the Clinical Laboratory Standards Institute [64,65]. Microbial suspensions corresponding to 1.5 × 10^8^ CFU/mL were prepared from 24 h cultures on a specific medium with agar. A stock of NPs (10 μg/μL) suspension prepared with sterile physiological buffer saline (PBS) was used. Petri plates with the medium were seeded with inoculums, and 10 µL of each sample was spotted. After diffusion, the dishes were incubated at 37 °C for 24 h.

#### 4.12.4. Quantitative Evaluation of Antimicrobial Activity

The minimum inhibitory concentration (MIC) assay used an adapted binary serial microdilution standard assessment in NB medium [64]. In a 96-well plate, for each NP sample, serial two-fold micro-dilutions were performed in 150 µL of broth medium seeded with the standard inoculum. The plates were incubated at 37 °C for 24 h. Visual and spectrophotometric analyses determined the MIC values by measuring the absorbance at 620 nm using the BIOTEK SYNERGY-HTX ELISA multi-mode reader (Winooski, VT, USA) [64].

#### 4.12.5. Semiquantitative Assessment of Microbial Adherence to the Inert Substratum

Biofilm development on the inert substratum was determined using the same serial two-fold microdilution method [64]. After 24 h of incubation and MIC measurements, the medium from the plates was removed, the walls were washed three times with PBS, and the bacterial cells adhered to the walls were fixed with methanol and tinted with 1% crystal purple. The dyed biofilm was resuspended with 33% acetic acid, and the absorbance was measured at 490 nm [64].

### 4.13. Statistical Analysis

The data results were statistically analyzed using GraphPad Prism, version 10.4, from GraphPad Software (San Diego, CA, USA). All experiments were performed in three independent determinations. The results are expressed as ±SD (standard deviation) and analyzed using a one-way analysis of variance (one-way ANOVA) followed by a multiple comparisons assay according to the experimental method. The differences between groups/samples were considered statistically significant when the *p*-value was <0.05.

## 5. Conclusions

Using orange peel extract as a multipurpose reducing, chelating, and stabilizing agent, this work offers a standardized, repeatable, and environmentally friendly production method for MgO NPs. Our approach uses the synergistic interaction of terpenoids, polymethoxyflavones, fatty acids, and sugars to precisely control crystallite size (~9 nm), enhance crystallinity, and direct particle morphology, in contrast to earlier green syntheses that relied on single phytochemical classes or inconsistent reaction parameters. As demonstrated by TGA-FTIR, XRD, and GC-MS, thorough characterization demonstrated that the orange peel extract actively contributes to precursor transformation rather than merely acting as an inert reaction medium. With a distinctive metabolic modulation effect in osteoblast-like cells and consistently high fibroblast and osteoblast-like cell viability (>80% at all tested concentrations), the resultant MgO NPs show promise for bone tissue engineering and wound healing applications where controlled cellular activity is beneficial.

Because of their cubic crystalline structure, surface charge, and nanoscale dimensions, biological experiments demonstrated moderate, broad-spectrum antibacterial and antibiofilm activity that was particularly effective against *P. aeruginosa* and *Gram-positive bacteria*. This dual-functional profile, which is uncommon in green-synthesized metal oxide NPs, is provided by the balance between excellent cytocompatibility and moderate antibacterial potential. Green nanomaterial synthesis is evolving from empirical trial and error and toward a logical, structure–activity-driven design process as a result of this study’s integration of mechanistic knowledge, complex structural analysis, and thorough biological evaluation. The results show how waste-derived phytochemical diversity can be strategically utilized to produce customized nanoparticle performance, in addition to positioning MgO NPs as promising candidates for medicinal applications.

## Figures and Tables

**Table 1:** Bioactive compounds found in orange peel extract.

Class	Details
Terpenoids	Compounds: Linalool, α-Terpineol, Carvone, Nootkatone Functional Role: Electron donation (reduction), chelation, antioxidant protection, surface stabilization Effect on NP: Facilitates metal ion reduction, controls nucleation, prevents oxidation, and minimizes agglomeration
Polymethoxyflavones	Compounds: Tangeretin, Nobiletin, Heptamethoxyflavone Functional Role: Redox activity, chelation, morphology optimization Effect on NP: Initiates oxide formation, promotes size and shape uniformity
Fatty Acids	Compounds: Palmitic, Linoleic, Oleic, Stearic Acid Functional Role: Capping, micelle formation, colloidal stability, adjusting of surface energy Effect on NP: Enhances dispersion, controls particle growth and morphology
Amino Acids and Sugars	Compounds: Derivatized forms Functional Role: Morphology optimization during synthesis Effect on NP: Influences shape and size distribution

**Table 2:** Zeta potential of green-synthetized MgO NPs.

Sample	Zeta Potential (mV)
MgO NPs	11.74 ± 0.49

## Data Availability

The original contributions presented in this study are included in the article/Supplementary Materials. Further inquiries can be directed to the corresponding authors.

## Supplementary Materials

The following supporting information can be downloaded at: https://www.mdpi.com/article/10.3390/ijms26189021/s1.
