# Supplementary material for: Green-Synthesized MgO Nanoparticles: Structural Insights and Antimicrobial Applications
Source: Int J Mol Sci. 2025 Sep 16;26(18):9021. doi: 10.3390/ijms26189021 (PMC12469372; doi:10.3390/ijms26189021)
Supplement: Supplementary file 1 [file ijms-26-09021-s001.zip › ijms-3867427-supplementary.pdf]

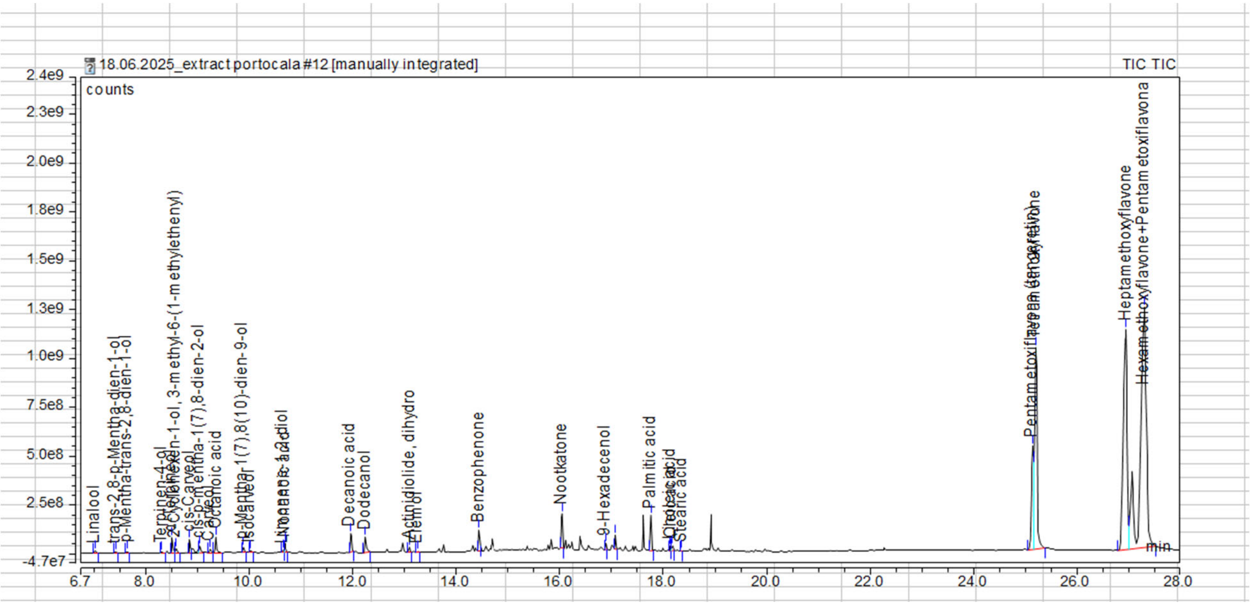


**Figure S1**. GC-MS chromatogram of orange peel extract showing identified bioactive compounds used in MgO nanoparticle synthesis.
